# Serum procalcitonin in bacterial & non-bacterial meningitis in children

**DOI:** 10.1186/s12887-018-1314-5

**Published:** 2018-11-02

**Authors:** Shipra Chaudhary, Nisha Keshary Bhatta, Madhab Lamsal, Rajendra Kumar Chaudhari, Basudha Khanal

**Affiliations:** B P Koirala Institute of Health Sciences, Dharan, Nepal

**Keywords:** Procalcitonin, Bacterial meningitis, Leukocyte count, Cerebrospinal fluid

## Abstract

**Background:**

Bacterial meningitis is a paediatric emergency with high mortality and morbidity requiring prompt diagnosis and treatment. Clinically, it is often difficult to differentiate between bacterial and non-bacterial meningitis. Several studies have demonstrated the raised values of serum procalcitonin (PCT) in bacterial infections including meningitis but without definite cut-off guidelines. Hence, this study was done to evaluate serum PCT as a marker to differentiate bacterial and non-bacterial meningitis in children and assess its efficacy.

**Methods:**

It was a cross-sectional study done over a period of 5 months (Aug 2016-Dec 2016) in the department of Paediatrics, B P Koirala Institute of Health Sciences (BPKIHS). Fifty children aged 3 months to 15 years with suspected meningitis were enrolled and investigated with relevant investigations like complete blood counts, and cerebrospinal fluid (CSF) analysis along with serum PCT. Patients were classified into bacterial (22) and non-bacterial meningitis (28) according to clinical & CSF findings and data analysed using SPSS software.

**Results:**

Serum PCT levels were significantly higher in bacterial meningitis group (median = 2.04 (1.2–3.18) ng/ml) compared with non-bacterial meningitis (median = 0.35 (0.18–0.35) ng/ml); *p* < 0.001. The sensitivity and specificity of serum PCT in diagnosis of bacterial meningitis at cut-off level of 0.5 ng/ml were 95.45% and 84.61% respectively. Procalcitonin showed maximum area under receiver operating characteristics (ROC) curve 0.991 (0.974–1.00) (*p* < 0.001) compared to total leukocyte count and CSF cytochemistry.

**Conclusion:**

Serum PCT has high sensitivity and specificity for early diagnosis of bacterial meningitis in children. Hence it can be a useful adjunct in differentiating bacterial and non-bacterial meningitis for prompt and better management of the children.

## Background

Meningitis is one of the most common central nervous system (CNS) infections encountered in infants and children. The source of infection in meningitis can be bacterial, viral, fungal, or parasitic in origin [1]. Bacterial meningitis is a paediatric emergency with high mortality and morbidity. Hence it must be diagnosed and treated promptly. But the similar clinical presentation often makes it difficult to differentiate bacterial and non-bacterial aetiologies in children [2, 3].

Currently, cerebrospinal fluid (CSF) analysis has been considered the gold standard for diagnosing bacterial meningitis, along with biomarkers like C-reactive protein (CRP) and white blood cell count (WBC) [2]. However, clinical criteria, gram staining, and bacterial antigen testing of CSF as well as the classic biological markers in blood (CRP, WBC, and neutrophil count) or CSF (protein level, glucose level, WBC, and neutrophil count) used alone do not offer 100% sensitivity and specificity for distinguishing bacterial and non-bacterial meningitis [2, 4]. Moreover, two or more days of waiting is required to identify bacterial growth in CSF cultures [4, 5].

After intensive research for new and rapid diagnostic methods for differential diagnosis of meningitis, serum procalcitonin (PCT) has been demonstrated to be the best marker for diagnosis of sepsis that also correlates with the extent and severity of microbial invasion [6]. Procalcitonin is now considered to be clinically most useful and superior to CRP due to its high diagnostic accuracy in various infectious pathologies, including sepsis, acute infectious endocarditis, and pancreatitis [7].

Procalcitonin, a 116-amino-acid peptide, which undergoes posttranslational proteolysis into calcitonin, is synthesized in C cells of the thyroid gland and secreted from leukocytes of the peripheral blood. The level of PCT increases (without an increase in calcitonin level) in the presence of bacterial lipopolysaccharides and cytokines that are associated with severe bacterial infections [8]. In contrast to this, there is minimal rise of PCT in patients infected with viruses [9]. Moreover, there is dramatic and quick increase in serum PCT levels after a single endotoxin injection [9, 10]. This type of response to a bacterial stimulus makes PCT level a potentially sensitive marker of severe bacterial infections including meningitis.

Several studies have demonstrated the raised values of serum PCT in bacterial infections including meningitis [11]. Studies from adults have shown PCT as a powerful diagnostic test for the assessment of suspected meningitis, allowing rapid differentiation between bacterial and non-bacterial (mostly viral) aetiologies, and earlier initiation of appropriate and necessary therapies. The evidence suggests that while serum PCT offers a similar specificity to the traditionally used CSF markers of meningitis, it also confers a higher sensitivity allowing for a more accurate overall diagnosis in patients with suspected meningitis. Hence the use of PCT serum assays in adult patients with suspected meningitis has been recommended but there is lack of recommendation in children [2]. The reported results of these studies are varied and a consensus has yet to be reached on the diagnostic value of PCT in meningitis. Hence, we evaluated serum PCT as a marker to differentiate bacterial and non-bacterial meningitis in children and assessed its efficacy in comparison to other investigations.

## Methods

This was a hospital-based cross-sectional study done in the department of Paediatrics, B P Koirala Institute of Health Sciences (BPKIHS), Nepal. Ethical clearance was obtained from institutional review committee. Fifty children aged 3 months to 15 years admitted during the study period (Aug 2016 - Dec 2016) with suspected meningitis were enrolled. Written informed consent was taken and those not giving consent, less than 3 months, more than 15 years, having secondary site of infection apart from meningitis or had received antibiotics for more than 3 days prior to admission were excluded. All the enrolled cases were worked-up and relevant diagnostic investigations like total/differential leukocyte count, CSF analysis by lumbar puncture were done. Along with these investigations, another 2 ml of blood was taken from each patient at the time of admission for serum PCT estimation which was performed on the fully-auto Chemiluminescence immunoassay (CLIA) analyzer Maglumi 1000 from Snibe Diagnostics with detection range of 0.13-100 ng/ml. Meningitis was diagnosed according to history, physical examination, CSF laboratory findings. It was further defined as bacterial meningitis if CSF showed bacteria in gram staining and/or culture, predominantly neutrophilic cells, glucose levels less than two-third of blood glucose or elevated CSF protein (> 45 mg/dl) despite receiving antibiotics for 1–3 days. Similarly, it was labelled as non-bacterial if CSF showed no growth of organisms, had predominantly lymphocytic counts, and normal (15-45 mg/dl) or slightly increased (up to 60 mg/dl) protein. The studied patients were categorized according to the CSF bacteriological and cytochemical profile into two groups: Group I (Bacterial meningitis) and Group II (Non bacterial meningitis). The details (demography, clinical features, investigation results and outcome) were recorded in a predesigned proforma. Data were analyzed using SPSS 17.0 statistical software using descriptive and analytic statistics (chi-square tests, independent sample t test, Mann Whitney U test) with significance set at 5%.

## Results

Amongst the 50 patients enrolled in this study, 22 patients fulfilled the criteria for bacterial meningitis and 28 patients non-bacterial meningitis. The median age of the studied population was 24(Interquartile range = 10.75–75) months and 60% were males. The common clinical presentations were fever (96%), convulsions (58%), and vomiting (40%). The demographic and clinical profile of the 2 groups were comparable with no statistically significant difference (Table 1).

**Table 1:** Demographic & clinical profile of patients (*n* = 50)

Characteristics	Group I Bacterial meningitis (*n* = 22)	Group II Non-bacterial meningitis (*n* = 28)	*P* value
Age (months)	23.5(11.75–66)	30 (9.25–81)	0.89 ^#^
Sex: Male Female	11(50%) 11(50%)	19 (67.85%) 9 (32.15%)	0.20*
Illness duration (days)	2.5 (1.75–4.5)	3 (2–3.75)	0.54 ^#^
Fever	21 (95.46%)	27 (96.43%)	0.36*
Convulsions	15 (68.18%)	14 (50%)	0.19*
Vomiting	6 (27.28%)	14 (50%)	0.10*
Headache	3 (13.64%)	7 (25%)	0.32*
Loss of consciousness	5 (22.72%)	2 (7.14%)	0.22*
Neck pain	8 (36.36%)	1 (3.57%)	0.06*
Decreased feeding	7 (31.81%)	7 (25%)	0.59*
Excessive crying	4 (18.18%)	3 (10.71%)	0.68*
GCS	12.86 ± 2.42	13.39 ± 2.18	0.40 ^##^
Neck stiffness	10 (45.45%)	6 (21.43%)	0.52*
Kernig’s sign	1 (4.54%)	3 (10.71%)	0.62*
Brudzinski sign	1 (4.54%)	3 (10.71%)	0.62*
Raised Intracranial pressure	5 (22.72%)	5 (17.86%)	0.67*
ICU need	5 (22.72%)	8 (28.57%)	0.64*
Mechanical ventilation	2 (9.09%)	5 (17.86%)	0.44*

(*Chi square test; ^#^ Mann Whitney U test; ^##^ Independent sample t test)

The cultured organisms from CSF were: *Staphylococcus aureus* (3), Acinetobacter sp. (3), Streptococcus pneumoniae (2), Enterococcus sp. (2), E coli (1). Eleven children had clinical presentation of meningitis but had received antibiotics for 1–3 days prior to admission. They were grouped as bacterial meningitis on the basis of other clinical features and CSF findings like leukocytosis with neutrophilic predominance, decreased glucose and/or elevated protein.

Serum PCT levels were significantly higher in bacterial meningitis group (median = 2.04 (1.2–3.18) ng/ml) compared to non-bacterial meningitis (median = 0.35 (0.18–0.35) ng/ml); *p* < 0.001.

Table 2 shows the comparison of the laboratory findings of blood and CSF between the two groups.

**Table 2:** Laboratory findings of blood and cerebrospinal fluid (*n* = 50)

Lab parameter	Group I Bacterial meningitis (*n* = 22)	Group II Non-bacterial meningitis (*n* = 28)	*P* value
Serum procalcitonin (PCT) (ng/ml)	2.86 ± 2.45	0.38 ± 0.24	< 0.001^#^
Total leukocyte count (TLC) per cubic milimetre (/mm^3^)	15,204.56 ± 8075.98	10,250 ± 4074.45	0.02^#^
Neutrophil (%)	53.45 ± 20.12	43.96 ± 15.43	0.06^##^
CSF			
TLC (/mm^3^)	28.59 ± 73.38	12.15 ± 18.55	0.427^#^
Neutrophil (%)	41.05 ± 35.08	21.23 ± 26.44	0.056^#^
Glucose (% of blood glucose)	42.18 ± 12.86	39.08 ± 14.79	0.75^##^
Protein (mg/dl)	72.86 ± 50.55	41.04 ± 11.51	0.001^#^

(^#^Mann Whitney U test; ^##^Independent sample t test)

The sensitivity and specificity of serum PCT was found to be 95.45% (77.16–99.88) and 84.6% (63.11–93.94) respectively with accuracy of 88% (75.69–95.47). Procalcitonin showed maximum area under receiver operating characteristics (ROC) curve 0.991 (0.974–1.00) (*p* < 0.001) compared to total leukocyte count and CSF cytochemistry.

Table 3 shows the efficacy of PCT (with diagnostic cut-off point > 0.5 ng/ml) compared to TLC (> 11,000/mm3), CSF TLC (> 10 cells/mm3), CSF neutrophil count (> 60%), CSF glucose (< 60% of blood glucose) and CSF protein (> 45 mg/dl) for diagnosis of bacterial meningitis.

**Table 3:** Efficacy of serum procalcitonin, total leukocyte count & cerebrospinal fluid cytochemistry in diagnosis of bacterial meningitis

Test	Sensitivity	Specificity	Area under curve (95% confidence interval)	*P* value
Procalcitonin	95.45%	84.6%	0.991 (0.974–1.00)	< 0.001
TLC	81.8%	50.0%	0.679 (0.523–0.835)	0.034
CSF				
TLC	54.5%	62.5%	0.566 (0.4–0.731)	0.438
Neutrophil	27.3%	88.0%	0.656 (0.496–0.817)	0.064
Glucose	90.9%	15.2%	0.537 (0.37–0.704)	0.664
Protein	68.2%	58.7%	0.788 (0.66–0.915)	0.001

## Discussion

Early diagnosis of meningitis and differentiation of bacterial from non-bacterial meningitis plays a vital role in the emergency management of children with suspected meningitis. Since Assicot et al. first proposed serum PCT as an early marker of bacteremia in 1993, there have been various descriptive reports on PCT [12]. Procalcitonin has been demonstrated to be ideal marker with highest accuracy for bacterial infections- allowing an early diagnosis, informing about the course and prognosis of the disease and facilitating therapeutic decisions [13, 14]. Procalcitonin has been found to be more specific and sensitive marker of various infections like respiratory tract infections, meningitis, acute infectious endocarditis and pancreatitis [7]. Different studies have found its sensitivity and specificity ranging from 50 to 100% but there is still variation in choice of the “abnormal” cut-off values due to diverse age range and nature of the study populations in those studies [5].

In this study we found serum PCT significantly higher in children with bacterial meningitis than non-bacterial meningitis. The mean PCT level in bacterial meningitis was 2.86 ± 2.45 ng/ml while that in non-bacterial meningitis was 0.38 ± 0.27 ng/ml (*p* < 0.001). This result is similar to that obtained by other investigators where PCT concentration increased in bacterial meningitis but not in viral meningitis and inflammatory diseases [3, 8, 9, 15–21].

This increase in serum PCT in bacterial meningitis can be explained by the increase of calcitonin gene (CALC-I gene) expression and release of PCT from all parenchymal tissues and differentiated cell types throughout the body in presence of bacterial lipopolysaccharides and cytokines associated with severe bacterial infections [8, 16].

In healthy subjects, circulating levels of PCT are very low, usually below 0.01 ng/ml and in viral infection and inflammation, the concentrations increase slightly but rarely above 1.0 ng/ ml. [18] Gendrel et al. have demonstrated elevated PCT levels (mean level, 54.5 mg/L; range, 4.8–110 mg/L) in bacterial meningitis while low levels (mean level, 0.32 mg/L; range, 0–1.7 mg/L; P. .0001) in viral meningitis in children when there is zone of overlapping values in CSF cells and proteins and CRP [9, 16]. Ibrahim et al. demonstrated cut off point > 0.5 ng/ml showing positive correlation for differentiating acute bacterial meningitis from non-bacterial origin [8].

It has long been known that leukocyte count can differentiate bacterial and non-bacterial infection including meningitis [3, 4]. Similarly CSF cytochemistry is used in ruling out meningitis cases [14]. In our study, TLC was significantly higher in bacterial meningitis but CSF parameters apart from protein had no significant difference between the two groups. This may be explained by lack of clear demarcation in cut-off for bacterial meningitis and also possible lysis of cells due to delayed processing or clearance with start of antibiotics in some cases.

This result is in agreement with other studies where leukocytosis was valuable in distinguishing between bacterial and non-bacterial infections but not an independent predictor of serious bacterial infection like PCT and CRP [8, 21–23]. In our study, the sensitivity and specificity of PCT (with diagnostic cut-off point > 0.5 ng/ml) were 95.45% (77.16–99.88) and 84.6% (63.11–93.94) respectively with accuracy of 88% (75.69–95.47) and maximum area coverage in receiver operative characteristic curve (ROC) 0.991 (0.974–1.00) (*p* < 0.001) as compared to TLC and CSF cytochemistry. Similarly, Usama M pointed out PCT concentration > 2 ng/ml had 100% sensitivity and negative predictive value but only 66% specificity and 68% positive predictive value for bacterial meningitis [5]. Procalcitonin has been found to have better specificity, sensitivity, predictive value and likelihood ratio than CRP, interleukin 6 and interferon-alpha in children for distinguishing between bacterial and viral infections in other studies from various parts of the world [2, 4, 8, 9, 17]. A meta-analysis showed serum PCT to be very accurate for rapid differentiation of the aetiology of paediatric meningitis with pooled sensitivity and specificity of 0.96 (95% CI = 0.92–0.98) and 0.89 (95% CI = 0.86–0.92) respectively [24]. CRP was not measured in our study but various studies have compared PCT with CRP and concluded the superiority of PCT over CRP in diagnosis.

Potential limitations of our study were that we could not measure serum PCT in follow-up due to the high cost of procalctionin kit. This would have helped to document the change in PCT with start of treatment for evaluation of treatment efficacy and further prognostication. Other studies have shown decrease of PCT over time with antibiotics treatment in bacterial meningitis [25].

Thus, serum PCT can act as a more sensitive and specific diagnostic tool in early differentiation of bacterial from non-bacterial meningitis in children and thereby help in better management of these children in emergency.

## Conclusions

Serum PCT has high sensitivity and specificity for early diagnosis of bacterial meningitis in children. Hence it can be a useful adjunct in differentiating bacterial and non-bacterial meningitis for prompt and better management of the children.

## Abbreviations

BPKIHS: B P Koirala Institute of Health Sciences
CLIA: Chemiluminescence immunoassay
CNS: Central nervous system
CRP: C reactive protein
CSF: Cerebrospinal fluid
CT: Computed Tomography
ELISA: Enzyme linked immunosorbent assay
G stain: Gram stain
IgM: Immunoglobulin M
IL: Interleukin
JE: Japanese encephalitis
MRI: Magnetic Resonance Imaging
PCT: Procalcitonin
PICU: Pediatric Intensive Care Unit
PMN: Polymorph
ROC: Receiver Operative Characteristic curve
TLC: Total leukocyte count
TNF: Tumor necrosis factor
WBC: White blood cell
ZN stain: Zeihl Neelsen stain
